# Thymic Epithelial Cell-Derived IL-15 and IL-15 Receptor α Chain Foster Local Environment for Type 1 Innate Like T Cell Development

**DOI:** 10.3389/fimmu.2021.623280

**Published:** 2021-03-01

**Authors:** Huishan Tao, Lei Li, Nan-Shih Liao, Kimberly S. Schluns, Shirley Luckhart, John W. Sleasman, Xiao-Ping Zhong

**Affiliations:** ^1^Department of Pediatrics-Allergy and Immunology, Duke University Medical Center, Durham, NC, United States; ^2^Institute of Molecular Biology, Academia Sinica, Taipei, Taiwan; ^3^Department of Immunology, University of Texas MD Anderson Cancer Center, Houston, TX, United States; ^4^Department of Entomology, Plant Pathology and Nematology, Department of Biological Sciences, University of Idaho, Moscow, ID, United States; ^5^Department of Immunology, Duke University Medical Center, Durham, NC, United States; ^6^Duke Cancer Institute, Duke University Medical Center, Durham, NC, United States

**Keywords:** IL-15, IL-15Rα, thymic epithelial cells, iNKT cells, γδT cells, type 1 innate like T cells

## Abstract

Expression of tissue-restricted antigens (TRAs) in thymic epithelial cells (TECs) ensures negative selection of highly self-reactive T cells to establish central tolerance. Whether some of these TRAs could exert their canonical biological functions to shape thymic environment to regulate T cell development is unclear. Analyses of publicly available databases have revealed expression of transcripts at various levels of many cytokines and cytokine receptors such as IL-15, IL-15Rα, IL-13, and IL-23a in both human and mouse TECs. Ablation of either IL-15 or IL-15Rα in TECs selectively impairs type 1 innate like T cell, such as *i*NKT1 and γδT1 cell, development in the thymus, indicating that TECs not only serve as an important source of IL-15 but also trans-present IL-15 to ensure type 1 innate like T cell development. Because type 1 innate like T cells are proinflammatory, our data suggest the possibility that TEC may intrinsically control thymic inflammatory innate like T cells to influence thymic environment.

How innate like T cell such as iNKT cell and γδT cell development is regulated and the role of thymic epithelial cells (TECs) in their development is not fully understood. We analyzed publicly available databases and have found that transcripts of many cytokines and cytokine receptors are expressed in both human and mouse TECs. We demonstrated that TEC-derived IL-15 and IL-15Rα play important and selective roles for type 1 innate like T cell, such as *i*NKT1 and γδT1 cell, development in the thymus. As iNKT1 cells are proinflammatory and contribute to adipogenesis, our data suggest the possibility that TEC may intrinsically control thymic inflammatory innate like T cells to influence thymic environment.

## Introduction

Two lineages of T cells, the αβT cell and γδT cell lineages that express distinct TCR receptor αβ chains and γδ chains, are generated in the thymus. αβT cells develop sequentially from the CD4^−^CD8^−^ double negative (DN) stage, the CD4^+^CD8^+^ double positive (DP) stage, and to the TCRαβ^+^CD4^+^CD8^−^ or TCRαβ^+^CD4^−^CD8^+^ single positive (SP) stage. Several αβT cells sublineages, including conventional CD4^+^ and CD8^+^ αβT cells, regulatory T cells, invariant natural killer T (*i*NKT) cells, and mucosal associate invariant T (MAIT) cells, with both distinct and common phenotypic and functional properties are evolved within the thymus (1–4). DN thymocytes can be sequentially defined into early T cell progenitors (ETP, Lin^−^cKit^+^CD44^+^CD25^−^), CD44^+^CD25^+^ DN2, CD44^−^CD25^+^ DN3, and CD44^−^CD25^−^ DN4 stages. At the DN2 and DN3 stages, γδT cells are generated after productively expressing functional γδ TCRs (5). In contrast to conventional αβT cells, *i*NKT cells, MAIT cells, and γδT cells can complete their differentiation into effector cells in the thymus, which appears to be regulated by thymic environment (6–11). These effector lineages include the type 1 sublineage (*i*NKT1/MAIT1/γδT1) that express T-bet and IFNγ, the type 2 sublineage (*i*NKT2/MAIT2/γδT2) that express Gata3 and IL-4, and the type 3 sublineage (*i*NKT17/MAIT17/γδT17) that express RORγt and IL-17A (8, 9, 12–19). While naïve T cells require several days to differentiate to effector cells, these innate like T cells can be activated quickly and are able to rapidly produce a variety of cytokines in response to agonistic stimuli to shape both innate and adaptive immunity.

In addition to crucial roles of TCR signals for both αβT and γδT cell development, local environment plays important roles in these innate like T cell maturation and differentiation to effector lineages. IL-15 is critical for development of *i*NKT cells, especially, for the NK1.1^+^CD44^+^ stage 3 and IFNγ-producing T-bet^+^ *i*NKT1 cells (20–23). Similarly, γδT cell effector lineages are also controlled by local cytokines. IFNγ-producing γδT1 cells are severely decreased in pLNs in IL-15 or IL-15Rα deficient mice. IL-15 induces γδT1 cell proliferation and survival via upregulating Bcl-xL and Mcl-1 (24, 25). An important feature of IL-15 signaling is that IL-15Rα serves as a high affinity IL-15-binding protein to *trans*-present IL-15 to the IL-15Rβ/γc complex on neighboring cells (26–30). IL-15Rα mediated *trans*-presentation of IL-15 promotes NK cells and CD8 T cell homeostasis (26–30). Interestingly, IL-15Rα deficiency causes severe impairment of stage 3 *i*NKT1 cell development (6, 7). Although it has been reported that radiation-resistant cells in the thymus provide IL-15 and *trans*-present IL-15 via IL-15Rα to promote *i*NKT cell development (6, 7), the exact cellular source of IL-15 and the cell type(s) that *trans*-present IL-15 via IL-15Rα have been unclear as the thymus contains many cell types including radiation resistant non-hematopoietic cells and some hematopoietic cells that could also be radiation resistant.

Thymic epithelial cells (TECs) are crucial for thymopoiesis and thymus function to generate a vast repertoire of T cells that are able to perform immune defenses but are also self-tolerated. Cortical TECs (cTECs) and medullary TECs (mTECs) localize in discrete regions in the thymus and perform different function (31–33). cTECs are mainly responsible for positive selection of developing thymocytes expressing functional TCRs capable of recognition of self-peptide/MHC complexes (34–37). mTECs ensure highly self-reactive T cells are ablated to establish central tolerance via presentation of promiscuously expressed tissue restricted antigens (TRAs) controlled by Aire and Fezf2 (34, 36, 38–41). In this report, we analyzed publicly available databases and revealed that TECs indeed express a variety of cytokine and cytokine receptors at various levels. We demonstrated further that ablation of either IL-15 or IL-15Rα in TECs selectively impaired development and/or homeostasis of *i*NKT1 and γδT1 cells in the thymus, indicating that TECs not only serve as an important source of IL-15 but also trans-present IL-15 to ensure type 1 innate like T cell development. Our data suggest that possibility that TEC may intrinsically control thymic inflammatory innate like T cells, which may in turn influence thymic environment.

## Results

### Expression of a Variety of Cytokines and Cytokine Receptors Including IL-15/IL-15Rα by mTECs

**Figure 1:**
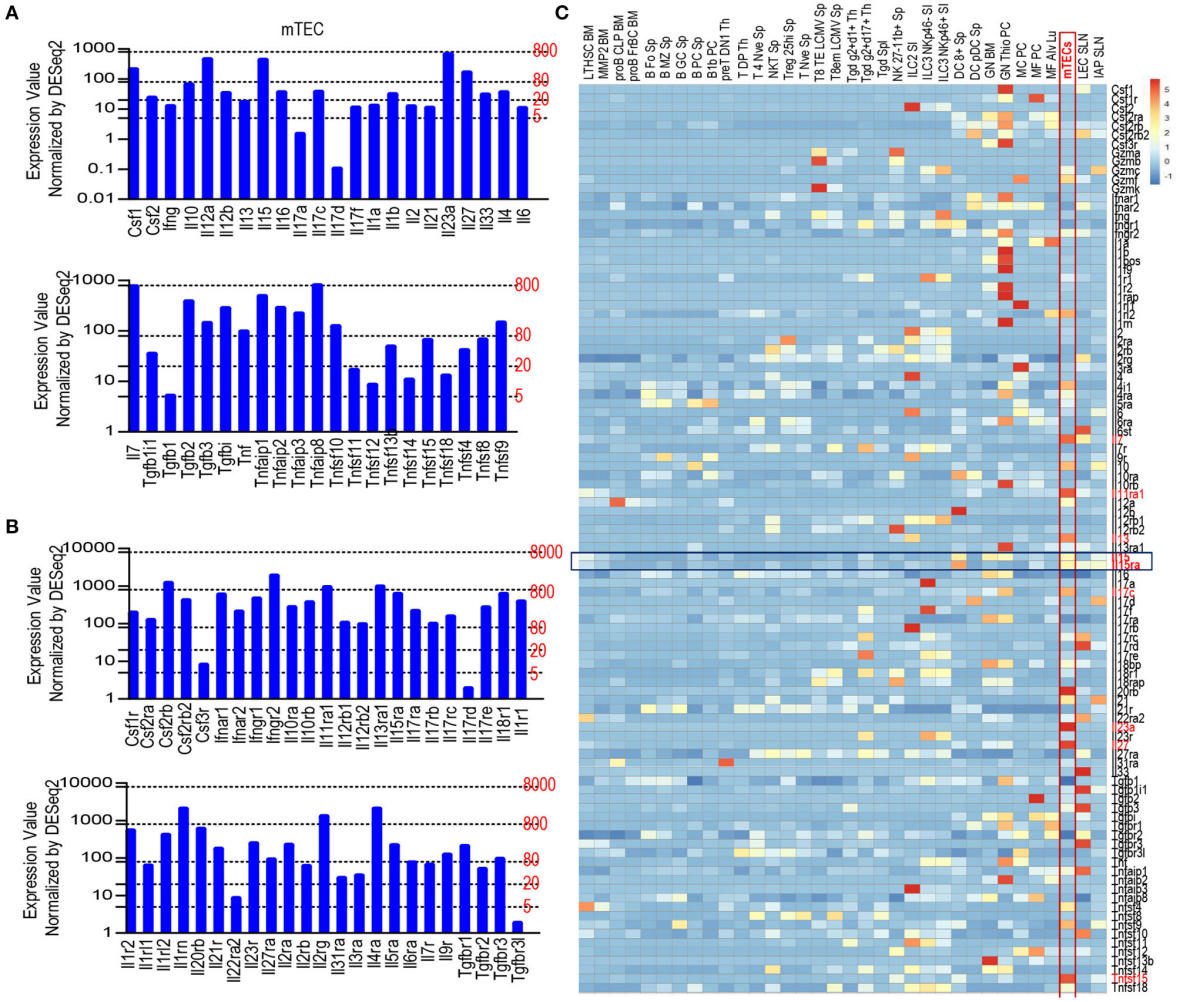
Expression of various cytokines and cytokine receptors in mTECs. **(A)** mRNA levels of cytokines in mTECs. Expression levels: 0–5, Trace; 2–20, very low; 20–80, low; 80–800, intermediate; 800–8,000, high according to Immgen.org. **(B)** mRNA levels of cytokine receptors in mTECs. **(C)** Heatmap showing relative mRNA levels of cytokine and cytokine receptor among mTECs, immune cells, and other stromal cells. Data shown are compiled from the RNAseq data from Immgen.org.

To determine the expression of cytokines and cytokine receptors in mTECs, we searched the publicly available Skyline RNAseq database from The Immunological Genome Project (Immgen.org) for mRNA levels in mTEC. mRNAs of many cytokines and their receptors could be detected in mTECs at various levels (Figures 1A,B). For cytokines, *Il7* is expressed at high levels and *Il23a* is expressed close to high levels (Figure 1A); *Csf1, IL12a, Il15, Il27, Tgfb2, Tgfb3, Tnf, Tnfsf9, and Tnfsf10* are expressed at intermediate levels; Many other cytokines such as *Il10, Il12b, il17c, Il1b, Il4, Il33*, and several Tnf superfamily members are expressed at low levels; several other cytokines such as *Ifng, Il17a, Il17d* and *Tgfb1*were expressed at very low or trace levels. For cytokine receptors, *Csf2rb, Ifngr2, Il11ra1, Il13ra1, Il1rn, Il2rg*, and *Il4ra* are expressed at high levels, whereas most cytokine receptors including *Il15ra* are expressed at intermediate levels and a few of cytokine receptors such as *Il22ra2, Csf3r*, and *Il17rd* were expressed between low and trace levels. Compared with different types of immune cells and other stromal cells, mTECs were among the highest expressers of mRNAs for multiple cytokines and cytokine receptors such as *Il7, Il10, Il11ra1, Il13, Il15, Il15ra, Il17c, Il20rb, Il23a, Il27, Tnfsf4, Tnfsf9*, and *Tnfsf15* (Figure 1C). Thus, mTECs express mRNAs of many cytokines and cytokine receptors at various levels.

### Expression of Discrete Cytokines in Murine TEC Subsets

**Figure 2:**
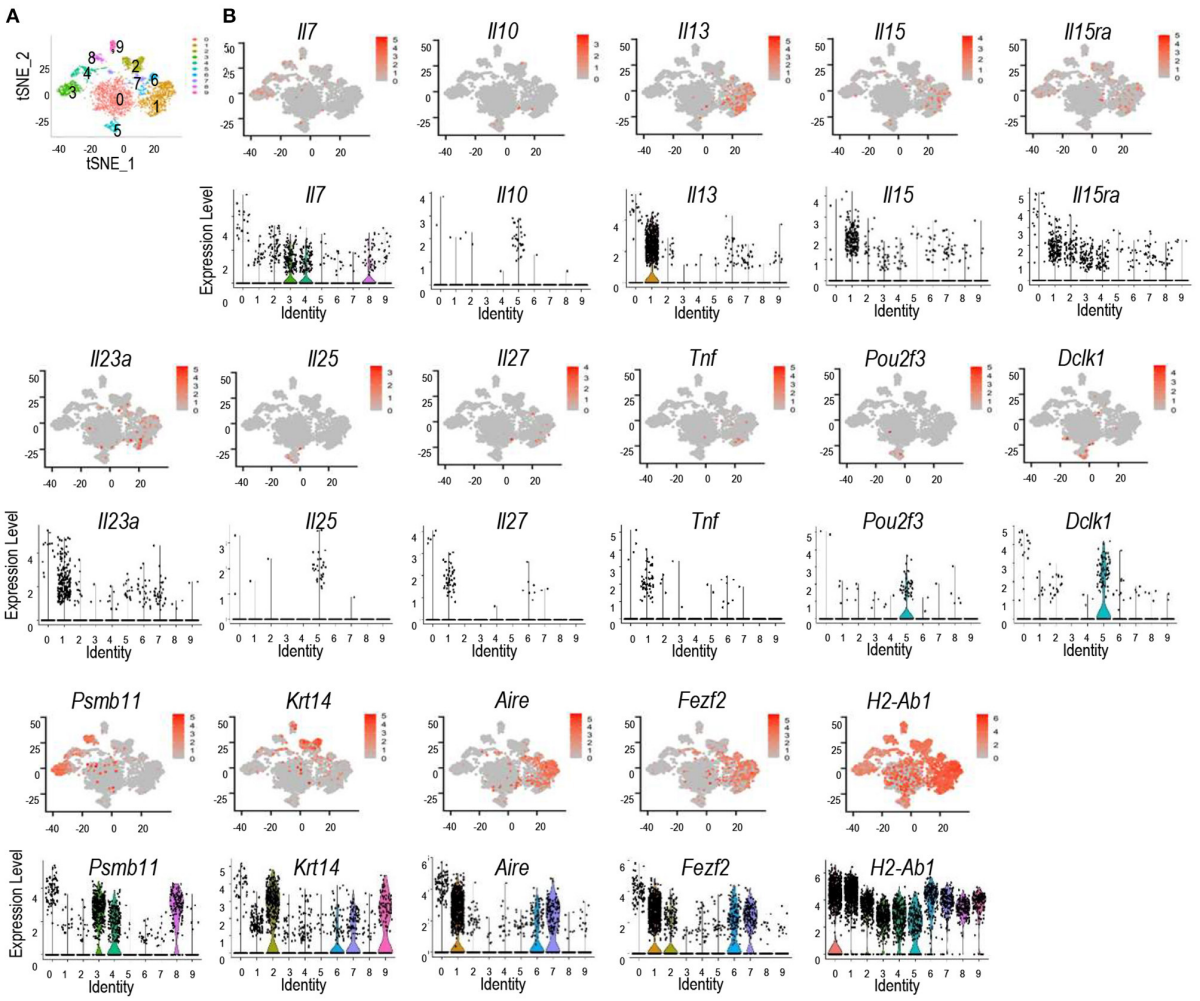
Discrete and promiscuous expression of cytokines and cytokine receptors in murine TEC subsets. scRNAseq data of TECs from 4 to 6 weeks old mice were analyzed. **(A)** tSNE plots showing TEC populations. **(B)** tSNE plots (top panels) and violin plots (bottom panels) showing distribution of cytokine/cytokine receptor expressing cells in different TEC populations. Data shown are generated from the scRNAseq data from Bornstein et al. (42).

Recently, murine TECs have been defined into 5 subsets based on single cell RNA sequencing analysis (42–48). To further investigate expression of cytokines and their receptors in TEC subsets, we analyzed scRNAseq data of TECs generated by the Ido Amit group, which had sequenced more TECs than other reports (42). Using the Seurat package approach (49), we could define TECs from 4 to 6 week old mice into 10 populations (Figure 2A). Populations 3, 4, and 8 are *Psmb11*^+^ and represent cTECs; populations 2 and 9 are *Krt14*^+^ and represent mTEC-I; populations 1, 6, and 7 are *Aire*^+^ and *Fezf2*^+^ and represent mTEC-II; population 5 is enriched with *Il25, Pou2f3*, and *Dclk1* and represents mTEC-IV or Tuft cells; population 0 is the most abundant population that expresses the highest levels of multiple molecules such as *H2-ab1, Psmb11, Krt14, Aire, Fezf2*, and *Dclk1* as well as cytokines and cytokine receptors, although at low frequencies. This population may represent mTEC-III (Figure 2B). Interestingly, *Aire*^+^*/Fezf2*^+^ populations 1, 6, and 7 (mTEC-II) also contain high levels and/or frequencies of cytokines/cytokine receptor mRNAs such as *Il13, Il23a, Il27*, and *Tnf*. In addition to *Il25*, mTEC-IV also is the highest *Il10* expresser. Although cTECs (populations 3, 4, and 8) contain highest frequencies of *Il7*^+^ cells, populations 1, 2, and 9 (mTEC-I/III) contain cells expressing higher levels of *Il7* than cTECs. *Il15* is expressed at high frequencies in population 1 and its levels appear higher in mTEC populations than cTEC populations, which is consistent with the detection of IL-15 reporter expression in the medulla in the mouse thymus (50). *Il15ra* is expressed at higher frequencies in populations 1 and 2 of mTECs and populations 3 and 4 of cTECs. However, the expression levels in these mTECs appear higher than in cTECs. Overall, *Aire/Fezf2*^+^ mTECs appear to express multiple cytokines at levels higher than cTECs while cTECs express higher levels of *Il7* than mTECs.

### Expression of Cytokines and Cytokine Receptors in Human TEC Subsets

**Figure 3:**
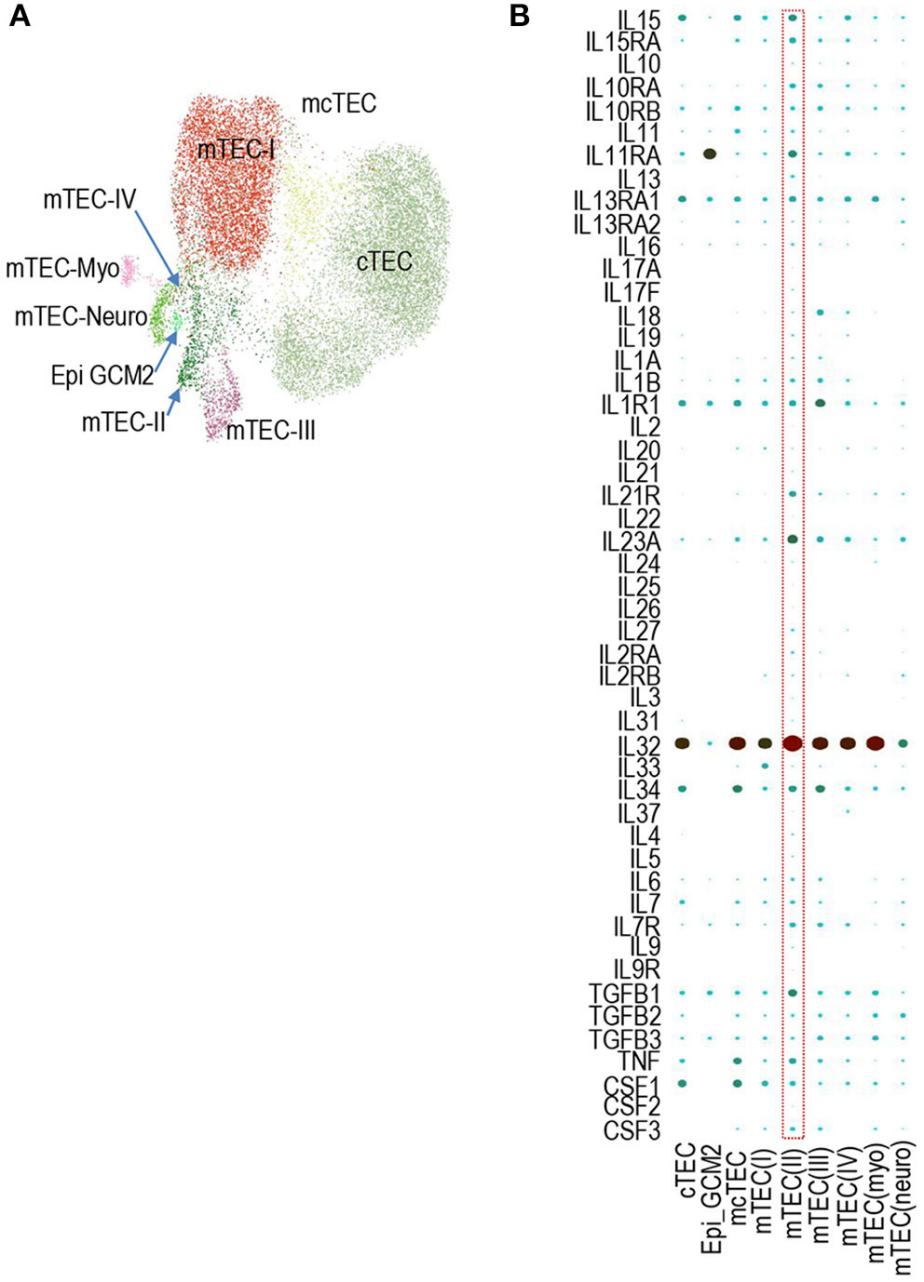
Expression of cytokines and cytokine receptors in human TEC subsets. **(A)** UMAP presentation of human fetal TEC clusters adapted from Jong-Eun Park et al. (51). **(B)** Dot-plot showing mRNA levels of indicated cytokine/cytokine receptors in the nine human TEC clusters from scRNAseq analysis. The size and color of the dot represent the percentage of cells within a cluster expressing the mRNA and the average expression level across all cells within a cluster. Light green and dark red represent low and high levels, respectively.

Similar to murine TECs, a recent report has found human TECs could also be defined into multiple populations based scRNAseq transcriptomic analysis (51). Human TECs also contain TEC-I – IV populations that mimic their murine counterparts. In addition, human TECs also contain MYOD1- and MYOG-expression myoid TEC-myo and NEUROD1- and NEURODG1- expressing TEC-neuro populations (Figure 3A) (51). We searched the Human Fetal Thymic Epithelium Gene Expression Web Portal (https://developmentcellatlas.ncl.ac.uk/datasets/HCA_thymus/human_epi/) for cytokines and cytokine receptors and revealed that human TECs also express many cytokine mRNAs at various levels (Figure 3B). *IL15, IL15RA, IL11RA, IL13RA1, IL1R1, IL23A, IL32, IL34, TGF1B1, TNF*, and *CSF1* are noticeably expressed at intermediate or high levels. Thus, similar to murine TECs, human TECs also expressed various cytokine/cytokine receptors at the mRNA levels.

### TEC-Derived IL-15 Promoted *i*NKT1 Development

**Figure 4:**
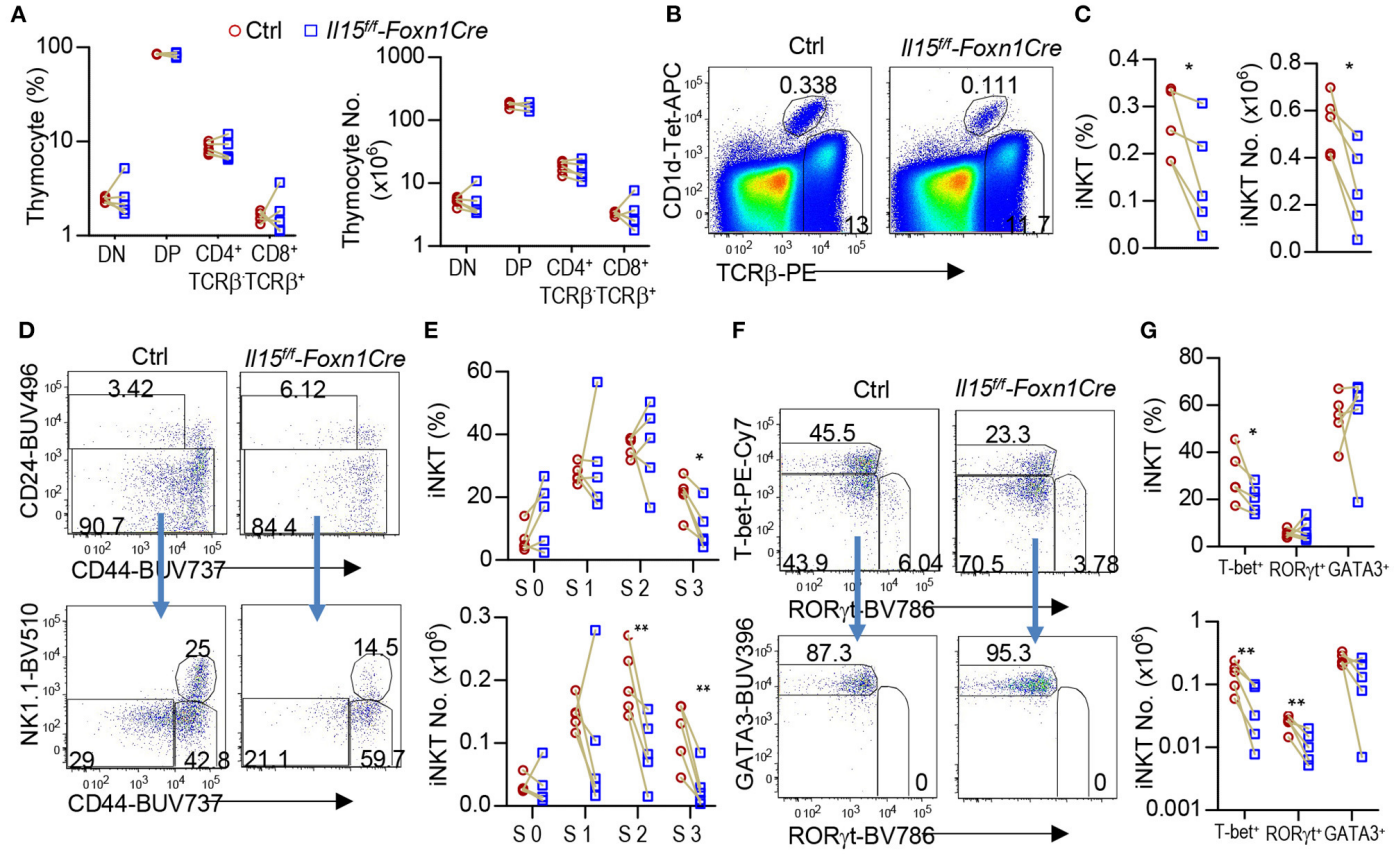
Impairment of *i*NKT1 development/homeostasis in TEC-specific IL-15 deficient mice. Thymocytes from 2 to 3 weeks old *Il15*^*f*/*f*^*-Foxn1Cre* and WT (*Il15*^+/+^*-Foxn1Cre* or *Il15*^*f*/*f*^) control mice were stained with fluorescently labeled PBS-57-loaded CD1d-Tetramer (CD1d-Tet), antibodies for TCRβ, CD4, CD8, other indicated molecules, and lineage markers (CD11b, B220, Ter119, CD11c, F4/80) as well as a fixable Live/Dead stain. **(A)** Scatter graph represents percentages and numbers of CD4^−^CD8^−^ double negative (DN), CD4^+^CD8^+^ double positive (DP), and TCRβ^+^ CD4^+^CD8^−^ or CD4^−^CD8^+^ single positive (SP) mature T cells. **(B)** Representative FACS plots showing TCRβ and CD1d-Tet staining of live gated Lin^−^ thymocytes. **(C)** Scatter plots of *i*NKT cell percentages and numbers. **(D)** Representative FACS plots showing CD24 vs. CD44 staining of total *i*NKT cells and CD44 vs. NK1.1 staining of CD24^−^ *i*NKT cells. **(E)** Scatter graphs of percentages and numbers of stage 0–3 *i*NKT cells. **(F)** Representative FACS plots showing T-bet vs. RORγt staining of CD24^−^ *i*NKT cells and GATA3 vs. RORγt staining of CD24^−^ T-bet^−^RORγt^−^ *i*NKT cells. **(G)** Scatter graphs of percentages and numbers of *i*NKT1/2/17 cells. Data shown are representative of or pooled from five experiments. Connection lines indicate sex-matched littermates. *p < 0.05; **p < 0.01 determined by two-tail pairwise Student *t*-test.

Thymic *i*NKT cells are defined into 0–3 stages based on differential expression of CD24, CD44, and NK1.1. IL-15/IL-15R signal promoted the development of T-bet^+^ *i*NKT1 cells, which occupy most of the CD44^+^NK1.1^+^ stage 3 *i*NKT cells (6, 7, 20–23). To investigate whether IL-15 expressed on TECs may exert biologic consequence besides serving as a TRA, we generated and analyzed TEC-specific IL-15 deficient, *Il15*^*f*/*f*^*-Foxn1Cre* mice. *Foxn1Cre* mice direct Cre expression starting on embryonic day 11.5 in TECs and ablate gene in both mTECs and cTECs (52). Compared with WT control mice, *Il15*^*f*/*f*^*-Foxn1Cre* mice did not show obvious alterations in thymocyte development (Figure 4A). However, their thymic *i*NKT cells, which were CD1d-Tetramer loaded with PBS-57 positive (CD1d-Tet^+^) and TCRβ^+^, showed 42.8 and 50.4% decreases of both percentages and numbers, respectively (Figures 4B,C). Within *i*NKT cells, CD24^+^CD44^−^ stage 0 and CD24^−^CD44^−^ stage 1 *i*NKT cells were not altered; CD24^−^CD44^+^NK1.1^−^ stage 2 *i*NKT cell percentages were not changed but numbers were decreased 54.8%; CD24^−^CD44^+^NK1.1^+^ stage 3 *i*NKT cells were decreased in both percentages (51.1%) and more severely in numbers (74.4%) (Figures 4D,E). Moreover, T-bet^+^RORγt^−^ *i*NKT1 cells were decreased in both percentages (31.8%) and, more severely, in numbers (66.2%). In contrast, T-bet^−^RORγt^+^ *i*NKT17 cell percentages were not decreased, although numbers of these cells were moderately decreased (54.9%). In contrast, T-bet^−^RORγt^−^Gata3^+^ *i*NKT2 cells were not altered in either percentages or numbers (Figures 4F,G). Thus, TEC-derived IL-15 is important for *i*NKT1 but not *i*NKT2 differentiation and/or homeostasis. Additionally, TEC-derived IL-15 also exerts a weak role for *i*NKT17 cell differentiation/homeostasis.

### IL-15Rα Expressed in TECs Selectively Promoted *i*NKT1 Cell Development

**Figure 5:**
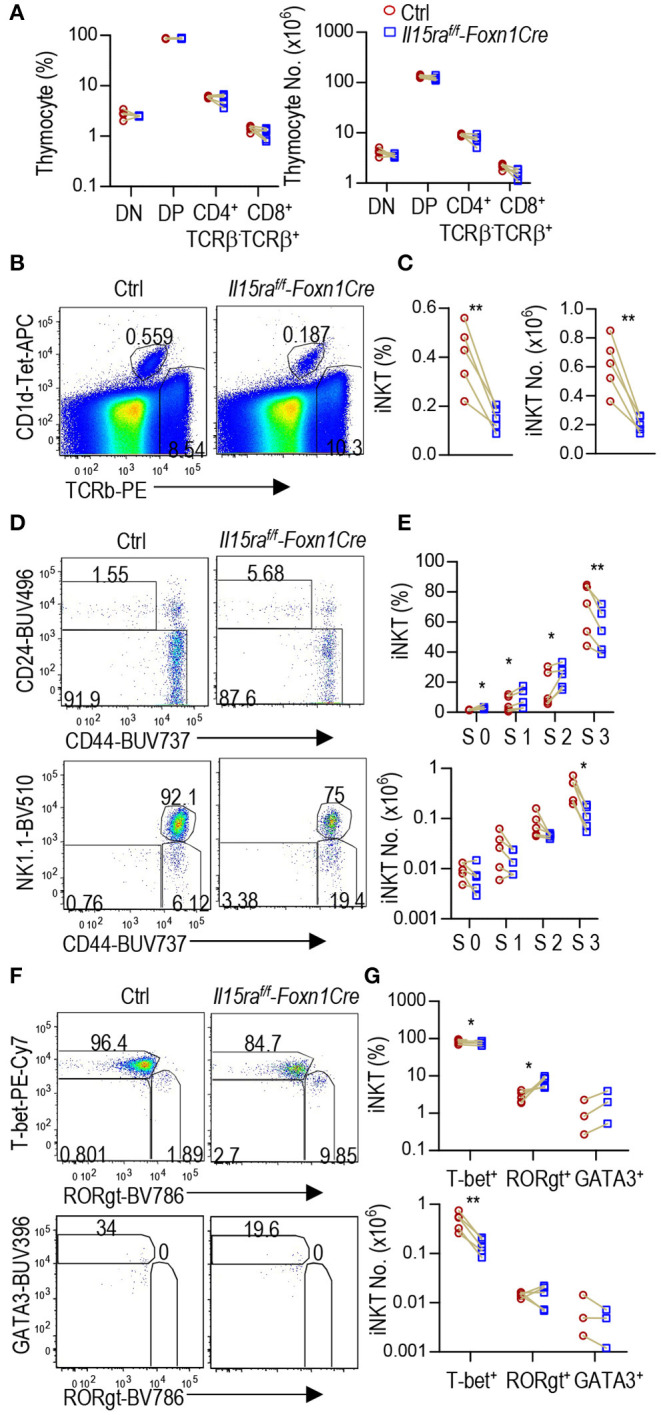
Selective defects in *i*NKT1 but not *i*NKT2/17 cell differentiation in *Il15ra*^*f*/*f*^*-Foxn1Cre* mice. Thymocytes from 6 to 8 weeks old *Il15ra*^*f*/*f*^*-Foxn1Cre* and WT (*Il15ra*^+/+^*-Foxn1Cre* or *Il15ra*^*f*/*f*^) control mice were analyzed similarly as Figure 4. **(A)** Scatter graph represents percentages and numbers of DN, DP, and TCRβ^+^ CD4^+^CD8^−^ or CD4^−^CD8^+^ SP mature T cells. **(B)** Representative FACS plots showing TCRβ and CD1d-Tet staining of live gated Lin^−^ thymocytes. **(C)** Scatter plots of *i*NKT cell percentages and numbers. **(D)** Representative FACS plots showing CD24 vs. CD44 staining of total *i*NKT cells and CD44 vs. NK1.1 staining of CD24^−^ *i*NKT cells. **(E)** Scatter graphs of percentages and numbers of stage 0–3 *i*NKT cells. **(F)** Representative FACS plots showing T-bet vs. RORγt staining of CD24^−^ *i*NKT cells and GATA3 vs. RORγt staining of CD24^−^ T-bet^−^RORγt^−^ *i*NKT cells. The gating of GATA3^+^ *i*NKT cells is based on its levels in T-bet^+^ *i*NKT cells. **(G)** Scatter graphs of percentages and numbers of *i*NKT1/2/17 cells. Data shown are representative of or pooled from three to five experiments. Connection lines indicate sex-matched littermates. *p < 0.05; **p < 0.01 determined by two-tail pairwise Student *t*-test.

IL-15Rα can *trans*-present IL-15 to IL-15R to trigger IL-15R signaling (26, 27). It has been reported that radiation-resistant thymic stromal cells may trans-present IL-15 to promote stage 3 and *i*NKT1 cell development via enhancing Bcl-2 mediated survival. The data were generated in lethally irradiated IL15Rα^−/−^ mice reconstituted with WT bone marrow cells (6, 7). However, these studies did not distinguish the role of TECs, other stromal cells, and radiation-resistant tissue resident macrophages or lymphoid tissue inducer cells. To investigate whether IL-15Rα expressed on TECs has biological consequences, we analyzed TEC-specific IL-15Rα deficient, *Il15ra*^*f*/*f*^*-Foxn1Cre* mice. Thymocyte development was not grossly affected in *Il15ra*^*f*/*f*^*-Foxn1Cre* mice (Figure 5A). However, *Il15ra*^*f*/*f*^*-Foxn1Cre* mice displayed 62.7 and 66.4% decreases of thymic *i*NKT cell percentages and numbers, respectively (Figures 5B,C). Within *i*NKT cells, percentages of stage 0, 1, and 2 cells were increased 2.1, 1.5, and 1.5-fold, respectively. However, their numbers were not significantly changed (Figures 5D,E). Stage 3 *i*NKT cells were decreased in both percentages (19.5%) and numbers (72.8%). Furthermore, T-bet^+^RORγt^−^ *i*NKT1 cells but not T-bet^−^RORγt^+^ *i*NKT17 or T-bet^−^RORγt^−^GATA3^+^ *i*NKT2 cells were severely decreased in *Il15ra*^*f*/*f*^*-Foxn1Cre* thymus (Figures 5F,G). Thus, IL-15Rα on TECs played an important and selective role for *i*NKT1 but not for *i*NKT2/17 differentiation or early *i*NKT cell development.

### IL-15 and IL-15Rα Expression in TECs Selectively Promoted γδT1 but Not γδT17 Cell Development

**Figure 6:**
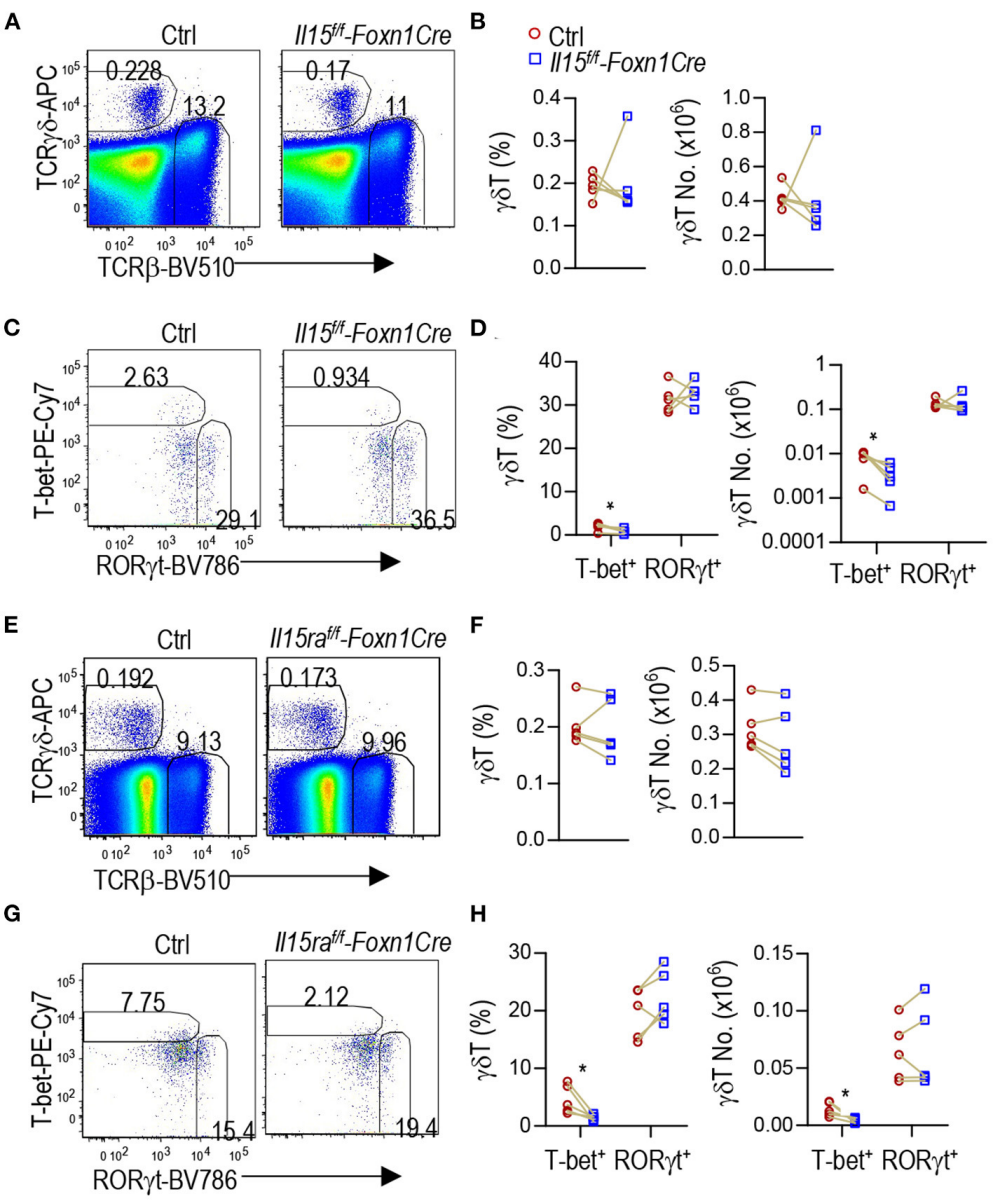
Selective defects in γδT1 but not γδT17 cell differentiation in TEC-specific IL-15 or IL-15Rα deficient mice. **(A–D)** Thymocytes from 2 to 3 weeks old *Il15*^*f*/*f*^*-Foxn1Cre* and WT (*Il15*^+/+^*-Foxn1Cre* or *Il15*^*f*/*f*^) control mice were labeled with fluorescently tagged antibodies as well as a fixable Live/Dead stain. **(A)** Representative FACS plots showing TCRβ and TCRγδ staining of live gated thymocytes. **(B)** Scatter graphs showing γδT cell percentages and numbers. **(C)** Representative FACS plots showing T-bet vs. RORγt in γδT cells. **(D)** Scatter graphs showing percentages and numbers of γδT1/17 lineages. Data shown are representative of or pooled from five experiments. Connection lines indicate sex-matched littermates. *p < 0.05 determined by two-tail pairwise Student *t*-test. **(E–H)** Thymocytes from 6 to 8 weeks old *Il15ra*^*f*/*f*^*-Foxn1Cre* and WT (*Il15ra*^+/+^*-Foxn1Cre* or *Il15ra*^*f*/*f*^) control mice were labeled with fluorescently tagged antibodies as well as a fixable Live/Dead stain. **(E)** Representative FACS plots showing TCRβ and TCRγδ staining of live gated thymocytes. **(F)** Scatter graphs showing γδT cell percentages and numbers. **(G)** Representative FACS plots showing T-bet vs. RORγt in γδT cells. T-bet^+^ γdT cell gating is based on its levels in TCRβ^+^CD44^−^CD122^−^ cells (Supplementary Figure 1B). **(H)** Scatter graphs showing percentages and numbers of γδT1/17 lineages. Data shown are representative of or pooled from five experiments. Connection lines indicate sex-matched littermates. *p < 0.05 determined by two-tail pairwise Student *t*-test.

γδT cells are another innate like T cell lineage that differentiate to effector lineages in the thymus. γδT cells also contain T-bet^+^ IFNγ-producing γδT1 and RORγt^+^ IL-17A-producing γδT17 lineages (53–55). γδT1 cells express CD122, the IL-2/15Rβ chain, and IL-15R signal is also important for γδT1 cell differentiation as well as γδT cell homeostasis and migration (20, 56–61). In *Il15*^*f*/*f*^*-Foxn1Cre* thymus, γδT cell percentages and numbers were not obviously different from controls (Figures 6A,B). However, T-bet^+^RORγt^−^ γδT1 cells but not T-bet^−^RORγt^+^ γδT17 cells were decreased 54.8% in percentages and 57.7% numbers (Figures 6C,D), indicating that TEC-derived IL-15 plays an important role for γδT1 cell development/homeostasis in the thymus.

Similarly, IL-15Rα deficiency in TECs in *Il15ra*^*f*/*f*^*-Foxn1Cre* mice did not obviously affect total γδT cell percentages or numbers (Figures 6E,F). However, γδT1 but not γδT17 cells in the thymus were decreased 69.1% in percentages and 70.4% numbers (Figures 6G,H). Thus, IL-15Rα on TECs also selectively promoted γδT1 cell differentiation but appeared dispensable for γδT17 differentiation.

## Discussion

It has been long appreciated that TECs control local environment to shape both conventional and innate like T cell development. We analyzed publicly available RNAseq and scRNAseq data and found that TECs, especially mTECs, express mRNAs for numerous cytokines and cytokine receptors such as *Il13, Il23a, Il15*, and *Il27* as well as *Il15ra* in mouse and/or human. Some cytokines and cytokine receptors including IL-15 and IL-15Rα are single chain molecules. It is conceivable that these molecules could be expressed as biologically functional molecules in TECs if they are properly processed inside these cells. While multiple previous studies have found radio-resistant cell derived IL-15 and/or IL-15Rα or have suggested that mTEC-derived IL-15 and/or IL-15Rα are important for iNKT cell, especially iNKT1 cell, development, no TEC-specific ablation of these molecules have been reported (6, 7, 62). We examined how TEC-specific IL-15 or IL-15Rα deficiency affects T cell, especially innate like T cell, development. We found that ablation of either IL-15 or IL-15Rα in TECs causes significant impairment of *i*NKT1 and γδT1 cell development in the thymus. Our data reveal that TECs not only serve as an indispensable source of IL-15 but also *trans*-present IL-15 for proper type 1 innate T cell development. At present, we do not known whether expression of various cytokine and cytokine receptors in TECs is dependent on Aire or Fezf2 and whether they function in TECs as TRAs to ensure T cell central tolerance. Nevertheless, our observations, together with those that mTEC-IV-derived IL-25 promotes *i*NKT2 development in the thymus (42, 43), suggest the possibility that some cytokines and cytokine receptors expressed in TECs may function both as TRAs and biologically active molecules that can exert their canonical biological functions in the thymus to shape local thymic environment to regulate T cell, particularly innate like T cell, development. Further studies are needed to examine whether TEC-specific ablation of IL-15 and IL-15Rα leads to escape the negative selection of T cells reactive to these molecules.

Of note, TEC-deficiency of IL-15 or IL-15Rα does not completely abolish type 1 innate like T cell development. It is possible other cell types such as dendritic cells and macrophages in the thymus may play partially redundant roles with TECs. Interestingly, TEC-specific IL-15 deficiency weakly reduced *i*NKT17 numbers in the thymus. This observation is consistent with previous reports that injection of IL-15/IL-15Rα complex induced expansion of both thymic iNKT1 and iNKT17 cells in mice (62, 63). Thus, TEC-derived IL-15 also plays an important role for *i*NKT17 cell development. Of note, our study does not distinguish the role of mTEC and cTEC derived IL-15/IL-15Rα for iNKT1 and γdT1 development as Foxn1Cre ablates genes in both mTECs and cTECs. However, IL-15 appears to be expressed mainly in mTECs and IL-15Rα is expressed at higher levels in mTECs than cTECs (Figure 2). Additionally, it has been found that mTECs are critical for iNKT1 cell development and induction of IL15R signaling by injecting IL-15/IL-15Rα complex into micer is able to overcome mTEC deficiency to promote iNKT1 development (62, 63). Similarly, γdT cells differentiate into effector lineages in the medulla (64). Together, these observations support that mTECs provide critical source of IL-15 for iNKT1 and γδT1 cell development.

Although mRNAs encoding many cytokines and cytokine receptors are expressed in TECs, some of them are biologically active only after complex with other molecules. For example, IL-12 and IL-23 that are heterodimers of an IL-12B (IL-12p40) subunit and the IL-12A (IL-12p35) subunit or the IL-23A (IL-23p19) subunit, respectively. Simultaneous expression of both subunits in the same cells would be required for formation of a functional protein. It is intriguing that expression levels among cytokines and cytokine receptors varies drastically in TECs. *Il23a* is expressed at the highest levels in mTECs. Whether such high levels of expression ensure full deletion of IL-23A reactive T cells, increase the chance of coexpression with IL-12B in some TECs, or IL-23A itself has biological activity in TECs remain to be explored.

The ability of TECs to produce cytokines and *trans*-presentation of cytokine(s) to shape thymic environment to control innate like T cell effector lineage differentiation/homeostasis in the thymus could have important implications for thymus biology. Despite the importance of the thymus for T cell generation, it undergoes involution or atrophy with advancing age. Thymic involution may contribute to the decline of immune functions, increased infection-induced mortality and morbidity, and autoimmune diseases in the elderly population (65–67). Although many extrinsic factors that can modulate the course of thymic involution have been identified, none is able to prevent or stop thymic involution. It has been noted that age-associated thymic involution is associated with accumulation of fatty tissue and inhibition of adipogenesis delays thymic involution. Interestingly, adipogenesis is promoted by local inflammation that is negatively controlled by *i*NKT2 and M2 macrophages but positively controlled by IFNγ and M1 macrophages (68–70). Given the ability of TEC sublineages to control type 1 and type 2 innate like T cell differentiation and *i*NKT cells can in turn regulate mTECs and thymic dendritic cells (63, 71), it is possible that thymic involution is an intrinsically programmed process encarved in and triggered by TECs (particularly mTECs) via shaping local thymic environment and presence of innate like T cell effector lineages in the thymus. A hypothesis warrants further investigation.

## Materials and Methods

### Mice

*Il15ra*^*f*/*f*^ mice (28) and *Il15*^*f*/*f*^ mice (72) were kindly provided by Drs. Kimberly Schluns and Averil Ma and Drs. Nan-Shih Liao and Shirley Luckhart, were bred with B6(Cg)-*Foxn1*^*tm*3(*cre*)*Nrm*^/J (*Foxn1Cre*) mice (52) that were kindly provided by Dr. Nancy Manley, to generate *Il15ra*^*f*/*f*−^*Foxn1Cre* and *Il15*^*f*/*f*−^*Foxn1Cre* mice as well as *Il15ra*^*f*/*f*^*, Il15*^*f*/*f*^, and *WT-Foxn1Cre* control mice. Mice were maintained in a pathogen free facility. All mouse experiments were performed following a protocol approved by the Institutional Animal Care and Use Committee of Duke University.

### Flow Cytometry and Antibodies

Thymocytes cells were prepared according to published protocols (73, 74). Cells were stained for surface markers with appropriate fluorochrome-conjugated antibodies and tetramers in PBS containing 2% FBS on ice for 30 min followed by intracellular staining of transcription factors using the eBioscience Foxp3 Staining Buffer Set according to the manufacturer's protocols. PE- or APC-labeled PBS-57-loaded CD1d-Tetramers (CD1d-Tet) were provided by the NIH Tetramer Core Facility. Fluorochrome-conjugated anti-TCRβ (clone H57-597), NK1.1 (clone PK136), CD44 (clone IM7), CD24 (clone M1/69), CD11b (clone M170), CD11c (clone N418), F4/80 (clone BM8), B220 (clone RA3-6B2), TER119/Erythroid Cells (clone TER-119), CD4 (GK1.5), CD8a (53-6.7), T-bet (4B10), TCRγδ (clone GL3), CD3 (clone 145-2C11), CD45 (clone 30-F11), CD27 (clone LG.3A10) were purchased from Biolegend; GATA3 (L50-823), RORγt (Q31-378) were purchased from BD Biosciences. Cell death was identified using the Live/Dead™ Fixable Violet Dead Cell Stain (Thermo Fisher Scientific). Data were collected using a BD LSRFortessa™ cytometer (BD Biosciences). Data were analyzed using the FlowJo Version 9.2 software (Tree Star).

### Expression of Cytokines and Cytokine Receptors From the Immunological Genome Project

Skyline RNAseq database from the Immunological Genome Project (Immgen.org) was searched for mRNA levels of indicated cytokines and cytokine receptors. In the Immunological Genome Project, 34 immune cell types from male and female mice were profiled by RNA-seq. Expression of mRNA was normalized for each cell types with the Z-score method. To visualize the different values among different cell types, the data for each cell were plotted as a heatmap using the pheatmap program (75).

### Analyses of Murine TEC scRNAseq Data

Raw counts of scRNAseq data of TECs from 4 to 6 weeks old mice reported by Bornstein et al. (42) were downloaded from GEO Database under the accession number GSE103967. scRNAseq data were pre-processed using the Seurat package (version 3.1.1) (49) in R (version 3.5.3). Genes expressed in fewer than 3 cells and cells with no more than 50 detected genes were filtered out. Filtered datasets were normalized the gene expression measurements for each cell by the total expression multiplied with a scale factor of 10,000 by default, followed by log-transformation of the results using the global-scaling normalization method, LogNormalize. The technical noise and/or biological sources of variation were mitigated via ScaleData function to improve downstream dimensionality reduction and clustering. Highly variable genes were screened with Find Variable Features function for downstream analysis. Principle component analysis (PCA) were performed on the scaled data using the RunPCA function. Significant PCs were identified as those with a strong enrichment of low *p*-value genes based on the Jackstraw algorithm. For cell clustering, k-nearest neighbors were calculated and the SNN graphs were constructed using Find Neighbors. Top 20 PCs were selected for analysis using Find Clusters. Cells within the graph-based clusters determined above were co-localized for visualization on the tSNE plot via RunTSNE and TSNEPlot. Find All Markers were applied to find markers that define clusters via differential expression. Feature Plot was applied to visualize individual gene expression on a tSNE plot. VlnPlot was applied to show expression probability distributions across clusters.

### Analyses of Human TEC scRNAseq Data

Expression of cytokines and cytokine receptors in human TECs was searched online based on scRNAseq analyses (https://developmentcellatlas.ncl.ac.uk/datasets/HCA_thymus/human_epi/) (51). Data were presented as a bubble plot with bubble size representing percentages of TECs expressing individual molecules and bubble color representing expression levels.

### Statistical Analysis

Data shown represent means ± SEMs and were analyzed with the two-tailed pairwise Student *t*-test using the Prism 5/GraphPad software for statistical differences. Each pair of mice represents sex-matched littermates and is indicated by a connecting line between test and control mice. *P*-values < 0.05 were considered significant (^*^p < 0.05, ^**^p < 0.01).

## Data Availability Statement

The raw data supporting the conclusions of this article will be made available by the authors, without undue reservation.

## Ethics Statement

The animal study was reviewed and approved by Institutional Animal Care and Use Committee of Duke University.

## Author Contributions

HT and LL designed and performed experiments, analyzed data, and participated manuscript preparation. N-SL, KS, and SL provided critical reagents and participated in manuscript preparation. X-PZ conceived the project, designed experiments, and wrote the manuscript. JS participated in manuscript preparation. All authors contributed to the article and approved the submitted version.

## Conflict of Interest

The authors declare that the research was conducted in the absence of any commercial or financial relationships that could be construed as a potential conflict of interest.

## References

[1] Sakaguchi S Yamaguchi T Nomura T Ono M. Regulatory T cells and immune tolerance. Cell. (2008) 133:775–87. 10.1016/j.cell.2008.05.009 18510923

[2] Yang W Gorentla B Zhong XP Shin J. mTOR and its tight regulation for iNKT cell development and effector function. Mol Immunol. (2015) 68:536–45. 10.1016/j.molimm.2015.07.022 26253278 PMC4679438

[3] Pan Y Deng W Xie J Zhang S Wan ECK Li L. Graded diacylglycerol kinases alpha and zeta activities ensure mucosal-associated invariant T-cell development in mice. Eur J Immunol. (2020) 50:192–204. 10.1002/eji.201948289 31710099 PMC7249235

[4] Chandra S Kronenberg M. Activation and function of iNKT and MAIT cells. Adv Immunol. (2015) 127:145–201. 10.1016/bs.ai.2015.03.003 26073984

[5] Hayday AC. Gammadelta T cell update: adaptate orchestrators of immune surveillance. J Immunol. (2019) 203:311–20. 10.4049/jimmunol.1800934 31285310

[6] Chang CL Lai YG Hou MS Huang PL Liao NS. IL-15Ralpha of radiation-resistant cells is necessary and sufficient for thymic invariant NKT cell survival and functional maturation. J Immunol. (2011) 187:1235–42. 10.4049/jimmunol.1100270 21709149

[7] Castillo EF Acero LF Stonier SW Zhou D Schluns KS. Thymic and peripheral microenvironments differentially mediate development and maturation of iNKT cells by IL-15 transpresentation. Blood. (2010) 116:2494–503. 10.1182/blood-2010-03-277103 20581314 PMC2953886

[8] Koay HF Gherardin NA Enders A Loh L Mackay LK Almeida CF. A three-stage intrathymic development pathway for the mucosal-associated invariant T cell lineage. Nat Immunol. (2016) 17:1300–11. 10.1038/ni.3565 27668799

[9] Legoux F Gilet J Procopio E Echasserieau K Bernardeau K Lantz O. Molecular mechanisms of lineage decisions in metabolite-specific T cells. Nat Immunol. (2019) 20:1244–55. 10.1038/s41590-019-0465-3 31431722

[10] Wang HX Cheng JS Chu S Qiu YR Zhong XP. mTORC2 in thymic epithelial cells controls thymopoiesis and T cell development. J Immunol. (2016) 197:141–50. 10.4049/jimmunol.1502698 27233961 PMC4912958

[11] Wang HX Shin J Wang S Gorentla B Lin X Gao J. mTORC1 in Thymic Epithelial Cells Is Critical for Thymopoiesis, T-Cell Generation, and Temporal Control of gammadeltaT17 Development and TCRgamma/delta Recombination. PLoS Biol. (2016) 14:e1002370. 10.1371/journal.pbio.1002370 26889835 PMC4758703

[12] Michel ML Mendes-da-Cruz D Keller AC Lochner M Schneider E Dy M. Critical role of ROR-gammat in a new thymic pathway leading to IL-17-producing invariant NKT cell differentiation. Proc Natl Acad Sci USA. (2008) 105:19845–50. 10.1073/pnas.0806472105 19057011 PMC2604995

[13] Watarai H Sekine-Kondo E Shigeura T Motomura Y Yasuda T Satoh R. Development and function of invariant natural killer T cells producing T(h)2- and T(h)17-cytokines. PLoS Biol. (2012) 10:e1001255. 10.1371/journal.pbio.1001255 22346732 PMC3274505

[14] Matsuda JL Zhang Q Ndonye R Richardson SK Howell AR Gapin L. T-bet concomitantly controls migration, survival, and effector functions during the development of Valpha14i NKT cells. Blood. (2006) 107:2797–805. 10.1182/blood-2005-08-3103 16357323 PMC1895373

[15] Coquet JM Chakravarti S Kyparissoudis K McNab FW Pitt LA McKenzie BS. Diverse cytokine production by NKT cell subsets and identification of an IL-17-producing CD4-NK1.1- NKT cell population. Proc Natl Acad Sci USA. (2008) 105:11287–92. 10.1073/pnas.0801631105 18685112 PMC2516267

[16] Wu J Yang J Yang K Wang H Gorentla B Shin J. iNKT cells require TSC1 for terminal maturation and effector lineage fate decisions. J Clin Invest. (2014) 124:1685–98. 10.1172/JCI69780 24614103 PMC3973110

[17] Chen Y Ci X Gorentla B Sullivan SA Stone JC Zhang W. Differential requirement of RasGRP1 for gammadelta T cell development and activation. J Immunol. (2012) 189:61–71. 10.4049/jimmunol.1103272 22623331 PMC3382004

[18] Shibata K Yamada H Sato T Dejima T Nakamura M Ikawa T. Notch-Hes1 pathway is required for the development of IL-17-producing gammadelta T cells. Blood. (2011) 118:586–93. 10.1182/blood-2011-02-334995 21606479

[19] Ribot JC deBarros A Pang DJ Neves JF Peperzak V Roberts SJ. CD27 is a thymic determinant of the balance between interferon-γ- and interleukin 17–producing γδ T cell subsets. Nat Immunol. (2009) 10:427–36. 10.1038/ni.1717 19270712 PMC4167721

[20] Kennedy MK Glaccum M Brown SN Butz EA Viney JL Embers M. Reversible defects in natural killer and memory CD8 T cell lineages in interleukin 15-deficient mice. J Exp Med. (2000) 191:771–80. 10.1084/jem.191.5.771 10704459 PMC2195858

[21] Matsuda JL Gapin L Sidobre S Kieper WC Tan JT Ceredig R. Homeostasis of V alpha 14i NKT cells. Nat Immunol. (2002) 3:966–74. 10.1038/ni837 12244311

[22] Ranson T Vosshenrich CA Corcuff E Richard O Muller W Di Santo PJ. IL-15 is an essential mediator of peripheral NK-cell homeostasis. Blood. (2003) 101:4887–93. 10.1182/blood-2002-11-3392 12586624

[23] Ranson T Vosshenrich CA Corcuff E Richard O Laloux V Lehuen A. IL-15 availability conditions homeostasis of peripheral natural killer T cells. Proc Natl Acad Sci USA. (2003) 100:2663–8. 10.1073/pnas.0535482100 12598649 PMC151397

[24] Corpuz TM Stolp J Kim HO Pinget GV Gray DH Cho JH. Differential responsiveness of innate-like IL-17- and IFN-gamma-producing gammadelta T cells to homeostatic cytokines. J Immunol. (2016) 196:645–54. 10.4049/jimmunol.1502082 26673141

[25] Colpitts SL Puddington L Lefrancois L. IL-15 receptor alpha signaling constrains the development of IL-17-producing gammadelta T cells. Proc Natl Acad Sci USA. (2015) 112:9692–7. 10.1073/pnas.1420741112 26195801 PMC4534247

[26] Lodolce JP Burkett PR Boone DL Chien M Ma A. T cell-independent interleukin 15Ralpha signals are required for bystander proliferation. J Exp Med. (2001) 194:1187–94. 10.1084/jem.194.8.1187 11602647 PMC2193508

[27] Dubois S Mariner J Waldmann TA Tagaya Y. IL-15Ralpha recycles and presents IL-15 In trans to neighboring cells. Immunity. (2002) 17:537–47. 10.1016/S1074-7613(02)00429-6 12433361

[28] Mortier E Advincula R Kim L Chmura S Barrera J Reizis B. Macrophage- and dendritic-cell-derived interleukin-15 receptor alpha supports homeostasis of distinct CD8+ T cell subsets. Immunity. (2009) 31:811–22. 10.1016/j.immuni.2009.09.017 19913445

[29] Mortier E Woo T Advincula R Gozalo S Ma A. IL-15Ralpha chaperones IL-15 to stable dendritic cell membrane complexes that activate NK cells via trans presentation. J Exp Med. (2008) 205:1213–25. 10.1084/jem.20071913 18458113 PMC2373851

[30] Burkett PR Koka R Chien M Chai S Boone DL Ma A. Coordinate expression and trans presentation of interleukin (IL)-15Ralpha and IL-15 supports natural killer cell and memory CD8+ T cell homeostasis. J Exp Med. (2004) 200:825–34. 10.1084/jem.20041389 15452177 PMC2213280

[31] Wendland K Niss K Kotarsky K Wu NYH White AJ Jendholm J. Retinoic acid signaling in thymic epithelial cells regulates thymopoiesis. J Immunol. (2018) 201:524–32. 10.4049/jimmunol.1800418 29848752

[32] Wang W Thomas R Sizova O Su DM. Thymic function associated with cancer development, relapse, and antitumor immunity - a mini-review. Front Immunol. (2020) 11:773. 10.3389/fimmu.2020.00773 32425946 PMC7203483

[33] Wang HX Pan W Zheng L Zhong XP Tan L Liang Z. Thymic epithelial cells contribute to thymopoiesis and T cell development. Front Immunol. (2019) 10:3099. 10.3389/fimmu.2019.03099 32082299 PMC7005006

[34] Klein L Kyewski B Allen PM Hogquist KA. Positive and negative selection of the T cell repertoire: what thymocytes see (and don't see). Nat Rev Immunol. (2014) 14:377–91. 10.1038/nri3667 24830344 PMC4757912

[35] Cowan JE Parnell SM Nakamura K Caamano JH Lane PJ Jenkinson EJ. The thymic medulla is required for Foxp3+ regulatory but not conventional CD4+ thymocyte development. J Exp Med. (2013) 210:675–81. 10.1084/jem.20122070 23530124 PMC3620359

[36] Perry JS Lio CW Kau AL Nutsch K Yang Z Gordon JI. Distinct contributions of Aire and antigen-presenting-cell subsets to the generation of self-tolerance in the thymus. Immunity. (2014) 41:414–26. 10.1016/j.immuni.2014.08.007 25220213 PMC4175925

[37] Coquet JM Ribot JC Babala N Middendorp S van der Horst G Xiao Y. Epithelial and dendritic cells in the thymic medulla promote CD4+Foxp3+ regulatory T cell development via the CD27-CD70 pathway. J Exp Med. (2013) 210:715–28. 10.1084/jem.20112061 23547099 PMC3620350

[38] Anderson MS Venanzi ES Klein L Chen Z Berzins SP Turley SJ. Projection of an immunological self shadow within the thymus by the aire protein. Science. (2002) 298:1395–401. 10.1126/science.1075958 12376594

[39] Takaba H Morishita Y Tomofuji Y Danks L Nitta T Komatsu N. Fezf2 orchestrates a thymic program of self-antigen expression for immune tolerance. Cell. (2015) 163:975–87. 10.1016/j.cell.2015.10.013 26544942

[40] Cosway EJ Lucas B James KD Parnell SM Carvalho-Gaspar M White AJ. Redefining thymus medulla specialization for central tolerance. J Exp Med. (2017) 214:3183–95. 10.1084/jem.20171000 28830910 PMC5679166

[41] Derbinski J Schulte A Kyewski B Klein L. Promiscuous gene expression in medullary thymic epithelial cells mirrors the peripheral self. Nat Immunol. (2001) 2:1032–9. 10.1038/ni723 11600886

[42] Bornstein C Nevo S Giladi A Kadouri N Pouzolles M Gerbe F. Single-cell mapping of the thymic stroma identifies IL-25-producing tuft epithelial cells. Nature. (2018) 559:622–6. 10.1038/s41586-018-0346-1 30022162

[43] Miller CN Proekt I von Moltke J Wells KL Rajpurkar AR Wang H. Thymic tuft cells promote an IL-4-enriched medulla and shape thymocyte development. Nature. (2018) 559:627–31. 10.1038/s41586-018-0345-2 30022164 PMC6062473

[44] Brennecke P Reyes A Pinto S Rattay K Nguyen M Kuchler R. Single-cell transcriptome analysis reveals coordinated ectopic gene-expression patterns in medullary thymic epithelial cells. Nat Immunol. (2015) 16:933–41. 10.1038/ni.3246 26237553 PMC4675844

[45] Kernfeld EM Genga RMJ Neherin K Magaletta ME Xu P Maehr R. A single-cell transcriptomic atlas of thymus organogenesis resolves cell types and developmental maturation. Immunity. (2018) 48:1258–70.e1256. 10.1016/j.immuni.2018.04.015 29884461 PMC6013397

[46] Miragaia RJ Zhang X Gomes T Svensson V Ilicic T Henriksson J. Single-cell RNA-sequencing resolves self-antigen expression during mTEC development. Sci Rep. (2018) 8:685. 10.1038/s41598-017-19100-4 29330484 PMC5766627

[47] Zeng Y Liu C Gong Y Bai Z Hou S He J. Single-cell RNA sequencing resolves spatiotemporal development of pre-thymic lymphoid progenitors and thymus organogenesis in human embryos. Immunity. (2019) 51:930–48.e936. 10.1016/j.immuni.2019.09.008 31604687

[48] Bacon WA Hamilton RS Yu Z Kieckbusch J Hawkes D Krzak AM. Single-cell analysis identifies thymic maturation delay in growth-restricted neonatal mice. Front Immunol. (2018) 9:2523. 10.3389/fimmu.2018.02523 30443254 PMC6221967

[49] Satija R Farrell JA Gennert D Schier AF Regev A. Spatial reconstruction of single-cell gene expression data. Nat Biotechnol. (2015) 33:495–502. 10.1038/nbt.3192 25867923 PMC4430369

[50] Cui G Hara T Simmons S Wagatsuma K Abe A Miyachi H. Characterization of the IL-15 niche in primary and secondary lymphoid organs in vivo. Proc Natl Acad Sci USA. (2014) 111:1915–20. 10.1073/pnas.1318281111 24449915 PMC3918838

[51] Park JE Botting RA Dominguez Conde C Popescu DM Lavaert M Kunz DJ. A cell atlas of human thymic development defines T cell repertoire formation. Science. (2020) 367:eaay3224. 10.1126/science.aay3224 32079746 PMC7611066

[52] Gordon J Xiao S Hughes B Su D-m Navarre SP Condie BG. Specific expression of lacZ and cre recombinase in fetal thymic epithelial cells by multiplex gene targeting at the Foxn1 locus. BMC Dev Biol. (2007) 7:69–9. 10.1186/1471-213X-7-69 17577402 PMC1906761

[53] Parker ME Ciofani M. Regulation of gammadelta T cell effector diversification in the thymus. Front Immunol. (2020) 11:42. 10.3389/fimmu.2020.00042 32038664 PMC6992645

[54] Papotto PH Reinhardt A Prinz I Silva-Santos B. Innately versatile: gammadelta17 T cells in inflammatory and autoimmune diseases. J Autoimmun. (2018) 87:26–37. 10.1016/j.jaut.2017.11.006 29203226

[55] Vantourout P Hayday A. Six-of-the-best: unique contributions of γδ T cells to immunology. Nat Rev Immunol. (2013) 13:88–100. 10.1038/nri3384 23348415 PMC3951794

[56] De Creus A Van Beneden K Stevenaert F Debacker V Plum J Leclercq G. Developmental and functional defects of thymic and epidermal V gamma 3 cells in IL-15-deficient and IFN regulatory factor-1-deficient mice. J Immunol. (2002) 168:6486–93. 10.4049/jimmunol.168.12.6486 12055269

[57] Hu MD Ethridge AD Lipstein R Kumar S Wang Y Jabri B. Epithelial IL-15 is a critical regulator of gammadelta intraepithelial lymphocyte motility within the intestinal mucosa. J Immunol. (2018) 201:747–56. 10.4049/jimmunol.1701603 29884699 PMC6075741

[58] Ma LJ Acero LF Zal T Schluns KS. Trans-presentation of IL-15 by intestinal epithelial cells drives development of CD8alphaalpha IELs. J Immunol. (2009) 183:1044–54. 10.4049/jimmunol.0900420 19553528 PMC2706935

[59] Shibata K Yamada H Nakamura R Sun X Itsumi M Yoshikai Y. Identification of CD25+ γδ T cells as fetal thymus-derived naturally occurring IL-17 producers. J Immunol. (2008) 181:5940–7. 10.4049/jimmunol.181.9.5940 18941182

[60] Pantelyushin S Haak S Ingold B Kulig P Heppner FL Navarini AA. Rorgammat+ innate lymphocytes and gammadelta T cells initiate psoriasiform plaque formation in mice. J Clin Invest. (2012) 122:2252–6. 10.1172/JCI61862 22546855 PMC3366412

[61] Lodolce JP Boone DL Chai S Swain RE Dassopoulos T Trettin S. IL-15 receptor maintains lymphoid homeostasis by supporting lymphocyte homing and proliferation. Immunity. (1998) 9:669–76. 10.1016/S1074-7613(00)80664-0 9846488

[62] White AJ Jenkinson WE Cowan JE Parnell SM Bacon A Jones ND. An essential role for medullary thymic epithelial cells during the intrathymic development of invariant NKT cells. J Immunol. (2014) 192:2659–66. 10.4049/jimmunol.1303057 24510964 PMC3948113

[63] Lucas B White AJ Cosway EJ Parnell SM James KD Jones ND. Diversity in medullary thymic epithelial cells controls the activity and availability of iNKT cells. Nat Commun. (2020) 11:2198. 10.1038/s41467-020-16041-x 32366944 PMC7198500

[64] Cowan JE Jenkinson WE Anderson G. Thymus medulla fosters generation of natural Treg cells, invariant gammadelta T cells, and invariant NKT cells: what we learn from intrathymic migration. Eur J Immunol. (2015) 45:652–660. 10.1002/eji.201445108 25615828 PMC4405047

[65] Masters AR Haynes L Su DM Palmer DB. Immune senescence: significance of the stromal microenvironment. Clin Exp Immunol. (2016) 187:6–15. 10.1111/cei.12851 27529161 PMC5167042

[66] Dixit VD. Impact of immune-metabolic interactions on age-related thymic demise and T cell senescence. Semin Immunol. (2012) 24:321–30. 10.1016/j.smim.2012.04.002 22546243

[67] Coder BD Wang H Ruan L Su DM. Thymic involution perturbs negative selection leading to autoreactive T cells that induce chronic inflammation. J Immunol. (2015) 194:5825–37. 10.4049/jimmunol.1500082 25957168 PMC4458423

[68] Hams E Locksley RM McKenzie AN Fallon PG. Cutting edge: IL-25 elicits innate lymphoid type 2 and type II NKT cells that regulate obesity in mice. J Immunol. (2013) 191:5349–53. 10.4049/jimmunol.1301176 24166975 PMC3847854

[69] Lynch L Nowak M Varghese B Clark J Hogan AE Toxavidis V. Adipose tissue invariant NKT cells protect against diet-induced obesity and metabolic disorder through regulatory cytokine production. Immunity. (2012) 37:574–87. 10.1016/j.immuni.2012.06.016 22981538 PMC4991771

[70] Lynch L Michelet X Zhang S Brennan PJ Moseman A Lester C. Regulatory iNKT cells lack expression of the transcription factor PLZF and control the homeostasis of T(reg) cells and macrophages in adipose tissue. Nat Immunol. (2015) 16:85–95. 10.1038/ni.3047 25436972 PMC4343194

[71] White AJ Lucas B Jenkinson WE Anderson G. Invariant NKT cells and control of the thymus medulla. J Immunol. (2018) 200:3333–9. 10.4049/jimmunol.1800120 29735644 PMC5985935

[72] Liou YH Wang SW Chang CL Huang PL Hou MS Lai YG. Adipocyte IL-15 regulates local and systemic NK cell development. J Immunol. (2014) 193:1747–58. 10.4049/jimmunol.1400868 25009203

[73] Shen S Wu J Srivatsan S Gorentla BK Shin J Xu L. Tight regulation of diacylglycerol-mediated signaling is critical for proper invariant NKT cell development. J Immunol. (2011) 187:2122–9. 10.4049/jimmunol.1100495 21775687 PMC3159847

[74] Shin J Wang S Deng W Wu J Gao J Zhong XP. Mechanistic target of rapamycin complex 1 is critical for invariant natural killer T-cell development and effector function. Proc Natl Acad Sci USA. (2014) 111:E776–83. 10.1073/pnas.1315435111 24516149 PMC3939904

[75] Ritchie ME Phipson B Wu D Hu Y Law CW Shi W. limma powers differential expression analyses for RNA-sequencing and microarray studies. Nucleic Acids Res. (2015) 43:e47. 10.1093/nar/gkv007 25605792 PMC4402510

