# Supplementary material for: Thymic Epithelial Cell-Derived IL-15 and IL-15 Receptor α Chain Foster Local Environment for Type 1 Innate Like T Cell Development
Source: Front Immunol. 2021 Mar 1;12:623280. doi: 10.3389/fimmu.2021.623280 (PMC7957058; doi:10.3389/fimmu.2021.623280)
Supplement: Supplementary file 1 [file Data_Sheet_1.PDF]

Figure S1

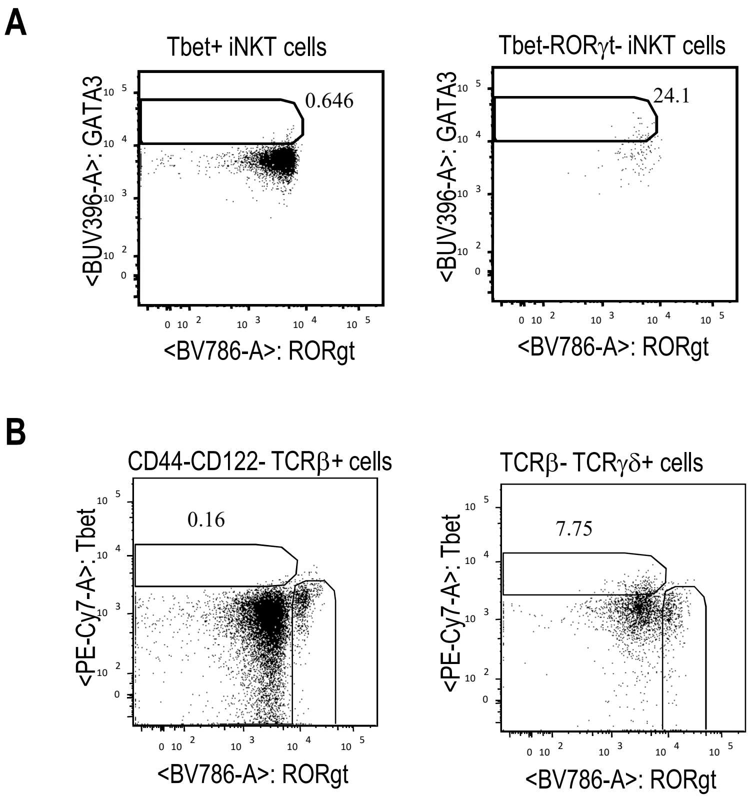

Figure S1. A. Gata3$^+$ cell gating in T-Bet$^+$ iNKT cells (left panel) and in Tbet$^-$ RORγt$^-$ iNKT cells (right panel) from WT thymus. **B.** Tbet$^+$ cell gating in CD44$^-$ CD122$^-$ TCRβ$^+$ cells (left panel) and TCRβ$^-$TCRγδ$^+$ cells (right panel) from WT thymus.
